# A State Medical Service: More Kite-Flying

**Published:** 1916-12-09

**Authors:** 


### A STATE MEDICAL SERVICE.

### More Kite-Flying.

"Our Medical Correspondent" in the *Times* has further elaborated his scheme for a universal State Medical Service. We are, it seems, "the victims of a bad and obsolete system." We do not, as we ought to do, regard our patients as heart cases, or lung cases, or fever cases, or throat and ear cases, requiring special treatment at the hands of a heart specialist, or a lung specialist, or a fever specialist, or a throat and ear specialist. We are so much the victims of a bad and obsolete system that we are apt to regard our patients as living human beings, as beings of an immensely complex organisation, whose parts are interdependent and correlated into one harmoniously working whole. Instead of regarding our patient thus, and pretending that he may become subject to disease, which may attack this or that organ, or several or many organs, the organ affected or most affected being a matter often of chance and often of minor importance, we ought to restrict our attention to the particular organ in which the disease is most conspicuous, and confine our treatment to that. Then we shall no longer be the victims of a bad and obsolete system, but shall be thoroughly up to date—and German.

Watertight Compartment Specialists.

In order that this desirable ideal may be attained, the general practitioner is to be abolished, and is to be replaced by a group of specialists, who are to "be paid salaries"—not, it will be observed, to receive salaries. Your penny-a-liner never employs the straightforward active voice if he can possibly distort his sentence into the passive. They are to be paid salaries by the State, and "left to arrange their work among themselves." Admirable notion! We know how paid Government officials arrange work among themselves, and how promptly it is executed in consequence. The present writer has recently received from a Government department an acknowledgment of a letter addressed to it only three months before. Under the present system a man who feels ill sends for his doctor, or goes to him, and is certain to be attended to within a very few hours at the utmost. Under the new system all this haste and hurry would be abolished. The sick man must first find out for himself what particular organ is diseased. He has, we will say, a tickling in his throat and a cough, so he goes to the throat specialist, who examines him and says, "You have come to the wrong shop, my friend. You have nothing the matter with your throat. You had better go to the local lung specialist." He accordingly goes to the lung specialist, who percusses him and auscultates him, and then says, "This is not my job. You are probably suffering from incipient tuberculosis. The tuberculosis specialist is the man for you." So off the patient goes to the tuberculosis specialist, who examines his sputum, and finds no bacilli. "Your cough," he says, "is probably a reflex cough. Better go to the stomach specialist and get him to examine you." So the patient starts off to see the stomach specialist; but he does not get there, for on the way the aneurysm of his aorta, which was pressing on his bronchus and so causing his cough, bursts, and kills him on the spot.

Competition for Work versus Competition for Dodging It.

"In any given small town," says "Our Medical Correspondent," "there will be found ten or a dozen doctors, all engaged in doing anything and everything, all competing bitterly against one another." The first part of this assertion is certainly not true. There are plenty of small towns or large villages in which there are only two or three doctors, and some in which there is only one; and under "Our Medical Correspondent's" scheme these two or three, or this one, would be abolished, and instead, a group of ten or a dozen specialists would be installed to do the work previously done by one or two or three general practitioners. It may be true that in some places a dozen doctors are all bitterly competing against one another, and it is to be presumed that "Our Medical Correspondent" would not make such an assertion unless he had some ground for it; but, granting that the assertion is true, is it so certain that the sick would be better off if the ten or a dozen doctors were all bitterly competing against one another to determine who should *not* attend the sick? If they bitterly compete in the one direction, would they not compete with equal bitterness in the other? The experience we have of Government departments gives us no assurance that they would not.

For many years the opinions of the *Times* on medical matters, advised as it was by Mr. Brudenell Carter, carried great weight, and were carefully attended to; and it is a great pity that this department of the leading and still great journal should have fallen under the guidance of an amateur, who is evidently quite ignorant of the conditions under which medical practice is carried on, and has a very limited and very erroneous knowledge of what constitutes disease.

